# Evaluating the Quality, Content Accuracy, and User Suitability of mHealth Prenatal Care Apps for Expectant Mothers: Critical Assessment Study

**DOI:** 10.2196/66852

**Published:** 2025-02-13

**Authors:** Fateme Asadollahi, Samira Ebrahimzadeh Zagami, Saeid Eslami, Robab Latifnejad Roudsari

**Affiliations:** 1 Department of Midwifery Student Research Committee Mashhad University of Medical Sciences Mashhad Iran; 2 Nursing and Midwifery Care Research Center Mashhad University of Medical Sciences Mashhad Iran; 3 Department of Medical Informatics School of Medicine Mashhad University of Medical Sciences Mashhad Iran; 4 Department of Midwifery School of Nursing and Midwifery Mashhad University of Medical Sciences Mashhad Iran

**Keywords:** pregnancy, prenatal care, mobile health apps, mHealth, women’s health, health care providers, quality assessment, content evaluation, suitability assessment, digital health, smartphones, eHealth, telehealth, telemedicine, health promotion, technology, functionality, systematic search

## Abstract

**Background:**

The proliferation of health apps in the digital health landscape has created significant opportunities for health promotion, particularly during pregnancy. However, despite the widespread distribution and popularity of pregnancy mobile apps, there are limited data on their quality and content.

**Objective:**

This study aimed to evaluate the quality, content accuracy, and suitability of the most popular and freely available Persian mobile health (mHealth) apps for prenatal care in expectant mothers.

**Methods:**

Through a systematic search, a total of 199 apps were screened from available app stores using the search term “pregnancy app” until July 2023. Inclusion criteria were apps in the Farsi language, freely available, downloaded more than 10,000 times, and designed for pregnant women. Ultimately, 9 apps met these criteria. These apps were downloaded onto mobile phones and assessed by 2 independent reviewers using the Mobile App Rating Scale (MARS), the Coverage and Depth of Information Checklist, and the Suitability Assessment of Materials (SAM). Statistical analyses explored relationships between app quality metrics and user ratings.

**Results:**

The 9 apps evaluated had an average MARS score of 3.55 (SD 0.61) out of 5. Aesthetics (mean 4.02, SD 0.45) and Functionality (mean 4.11, SD 0.36) scored the highest, followed by Engagement (mean 3.29, SD 0.53) and Information (mean 3.09, SD 0.48). User star ratings did not strongly correlate with MARS scores (*r*=0.38, *P*>.05). Regarding health information coverage, 6 out of 9 (66.7%) apps were rated as poor, and 3 (33.3%) as adequate. For SAM, 4 (44.4%) apps were rated as superior and 5 (55.6%) as adequate. No app received a poor score.

**Conclusions:**

The study underscores the need for improved standards in pregnancy app development to enhance educational efficacy and user satisfaction. Health care providers should recommend high-quality pregnancy apps with appropriate content to ensure effective health promotion. These findings contribute to understanding the current landscape of pregnancy apps and highlight areas for future research and regulatory attention.

**Trial Registration:**

PROSPERO CRD42023461605; https://www.crd.york.ac.uk/prospero/display_record.php?RecordID=461605

## Introduction

eHealth represents an innovative approach within the health care sector, using information and communication technology to enhance access to health care services and improve their quality [1]. eHealth encompasses various digital technologies, including telemedicine, mobile health (mHealth) apps (MHAs), electronic health records, and health information systems. These technologies aim to bridge the gap between health care providers and patients by enabling remote access to health care services, improving communication, and enhancing the overall quality of care [2].

MHAs are an integral part of the broader digital health ecosystem, including wearable technologies, virtual reality, telemedicine, and eHealth. These apps significantly enhance the accessibility and delivery of health services, especially with the increasing demand for smartphones and other digital devices driven by rapid technological advancements [3]. These apps empower individuals to participate in symptom control and identification, receive treatment, and obtain personal feedback and motivational support [2,4,5]. Pregnancy apps, in particular, have become popular resources for expectant mothers, offering functionalities ranging from fetal development tracking to health tips and educational content [6,7]. However, the reliability of health recommendations provided by pregnancy apps remains a significant concern. For instance, a study found that 89.7% of Chinese mobile apps for pregnancy and postpartum care did not provide safety statements or supporting evidence, and 68% of US apps similarly lacked evidence-based content [8]. Also, a systematic review of sexual and reproductive health apps revealed that, while a variety of apps exist, only a few meet high-quality design standards or demonstrate effectiveness in real-life settings [9]. These findings emphasize the critical need for research into the usability and evidence-based development of MHAs, particularly those targeting pregnancy care.

It is crucial for these apps to provide accurate and reliable evidence-based content that considers the cultural and linguistic needs of the target audience, including information on cultural practices and traditions related to pregnancy and childbirth [10].

While the quantity and user acceptance of Iranian pregnancy apps have grown significantly, the credibility of the information within these apps remains invalidated. A study conducted in Iran found that only 1.3% of pregnancy-related mobile apps were developed with the participation of obstetricians, and only 5% used reliable information resources [11]. This lack of professional input may affect the accuracy and reliability of the information these apps provide.

Despite the proliferation of pregnancy apps, there is a notable lack of research evaluating Persian-language apps. Existing studies on digital health tools in Persian often overlook the unique challenges faced by expectant mothers, such as the need for culturally relevant information and user-friendly interfaces that accommodate varying levels of comprehension and accessibility requirements specific to their needs [12]. Furthermore, data on the effectiveness of these apps in delivering evidence-based health information and supporting positive health outcomes is scarce [11].

The aim of this study is to assess MHAs for Iranian pregnant women by evaluating them across three key aspects: (1) quality assessment; (2) content accuracy via assessing coverage and depth of information, which assesses how thoroughly the app addresses relevant health topics, including work and rest practices during pregnancy, nutrition education, stress management, interpersonal relationships, and pregnancy care instructions, with significant implications for maternal health and well-being; and (3) user suitability of materials, which examines the quality of the app’s content to ensure it is accurate, reliable, and user-friendly. Specifically, this study seeks to answer the following questions:

1. What apps are available?

2. What is the quality of these apps, as measured by the Mobile App Rating Scale (MARS)?

3. How comprehensive is the content provided by these apps?

4. How suitable are these apps for expectant mothers based on their design and cultural relevance?

## Methods

### Study Design and Protocol Registration

This study used a systematic approach to identify, select, and evaluate Persian-language pregnancy apps available up to July 2023. The methodology was designed to ensure a rigorous and transparent evaluation process. A detailed protocol for the review was developed and registered with the International Prospective Register of Systematic Reviews (PROSPERO; ID CRD42023461605).

#### Search Strategy and Inclusion Criteria

The app search was conducted between June 1, 2023, and July 31, 2023, focusing on major platforms commonly used by Persian-language app users. Searches were performed on Google Play Store, Cafebazaar App Store, Myket Market, Kandoo, Iran Apps, Avval Market, and Pars Hub. To enhance comprehensiveness, an internet-based search via Google was also conducted as a supplementary measure to identify apps not listed on these platforms.

Given the dynamic nature of search results on the Google Play Store, the search was conducted manually to ensure relevance. The Apple App Store is not officially accessible in Iran due to regional restrictions. However, using a virtual private network is legal in Iran, and Iranian users frequently use virtual private networks to connect to the store and download apps. Apps were identified by sequentially navigating through search results. We screened all results until no new eligible apps were identified, which required reviewing up to 10 pages per platform. No web crawler was used, but search results were manually exported by recording app details (eg, name, description, and download count) directly into a predesigned data extraction form.

The search was conducted using a combination of Persian keywords related to pregnancy and their English equivalents. Search terms included were “pregnancy,” “prenatal care,” “motherhood,” “pregnant,” and “mother and baby.” Apps were considered eligible if they met the following criteria: the app must be in Persian, freely available, with or without in-app purchases, compatible with the Android operating system, having more than 10,000 downloads, designed for pregnant women, provided information on at least one of the following topics: work and rest practices during pregnancy, nutrition education, stress management, interpersonal relationships, or pregnancy care instructions.

Apps were excluded from the analysis if they met any of the following criteria: inaccessibility due to dead or broken links, duplication, design as e-books, news sources, magazines, podcasts, blogs, games, or gaming-related content. In addition, apps were excluded if their primary function was monitoring or timing without providing educational content, or if they required paid subscriptions or included freemium content that limited access to essential features.

#### Screening Process and Data Extraction

In order to determine eligibility, two independent reviewers (FA and SEZ) screened the titles, images, and descriptions of the identified apps during the search. In cases of disagreement, a third senior reviewer (RLR) was consulted to reach a consensus.

Data from eligible apps were extracted systematically by two reviewers (FA and SEZ) who were trained to ensure consistency and accuracy.

Eligible apps were downloaded and tested on a Xiaomi Mi Mix 3 device running Android 12. Extracted data included app name, version, developer, cost, in-app purchases, user rating, number of ratings, and last update date.

#### App Features and Quality Assessment

Using 3 primary assessment tools, 2 reviewers with expertise in midwifery and reproductive health independently evaluated the apps.

##### The MARS

MARS evaluates app quality across 4 dimensions including (1) engagement to assess fun, interest, adaptability, interactivity, and target group relevance; (2) functionality to examine performance, usability, navigation, and gestural design; (3) aesthetics to evaluate layout, graphics, and visual appeal; and (4) information quality to review the accuracy, goals, credibility based on the evidence and quality, and quantity of information, including visual information.

Apps were rated using a 5-point scale (1=inadequate to 5=excellent). A mean score was calculated to determine overall quality. Disagreements were resolved by involving a third assessor.

The validity of the Persian (Farsi) version of the MARS questionnaire, translated and culturally adapted from the original scale, was rigorously assessed and confirmed through various psychometric measures. The fit indices demonstrated strong construct validity for each dimension (root-mean-square error of approximation [RMSEA]=0.074, Tucker-Lewis index [TLI]=0.922, comparative fit index [CFI]=0.940, and standardized root-mean-square residual [SRMR]=0.059). Reliability was reported as good to excellent across domains, with Omega coefficients ranging from 0.79 to 0.93, indicating high internal consistency. Furthermore, the instrument exhibited strong interrater reliability, with an intraclass correlation coefficient of 0.82, demonstrating a high level of objectivity [13].

##### Coverage and Depth of Information Checklist

This researcher-developed tool assessed educational content based on guidelines by Iran’s Ministry of Health [14], which are provided in Multimedia Appendix 1. The checklist used in this study was developed and validated to ensure its reliability and suitability for evaluating MHAs designed for Iranian pregnant women. The development process began with an extensive review of the literature, expert consultations, and adherence to relevant maternal health guidelines to identify key topics and items for inclusion. These topics covered essential domains such as pregnancy care, stress management, nutrition education, and exercise practices during pregnancy. Coverage was scored as follows: correct and sufficient (2 points), partially correct or insufficient (1 point), and incorrect or not addressed (0 points). The total score categorized app content as Superior (41-46 points, 90%-100%), Adequate (23-40 points, 50%-89%), or Poor or Low (<23 points, ≤49%). The resulting checklist was structured with clear, measurable items to evaluate the quality, coverage, and depth of information provided by the apps.

To ensure the checklist was a reliable and effective evaluation tool, it underwent a pilot testing phase. A sample of 5 MHAs was selected for this pilot, chosen to represent a variety of features and content typically found in apps targeting pregnant women. Two independent reviewers with reproductive health specialists having experience in the evaluation of health apps assessed the apps using the checklist. This process served two primary purposes: to evaluate the internal consistency of the checklist items and to measure interrater reliability.

The checklist showed strong internal consistency (Cronbach α=0.85) and substantial interrater reliability (Cohen κ=0.80), confirming its alignment and consistency in measuring information coverage and quality. Minor ambiguities identified during pilot testing were revised, resulting in a robust and validated tool used to evaluate the MHAs comprehensively (Multimedia Appendix 2).

##### Suitability Assessment of Materials

The authors conducted a suitability assessment of patient education material using the Suitability Assessment of Materials (SAM) tool. Each item was rated as superior (2 points), adequate (1 point), or not suitable (0 points). The SAM consists of 22 items grouped under four categories: literacy demand, layout and type, learning stimulation and motivation, and cultural appropriateness. Apps featuring content that lacked cultural alignment, such as multimedia showcasing non-Iranian contexts or dietary advice incompatible with local practices, were found to be less effective in addressing user needs. In contrast, apps that included culturally tailored recommendations, such as adherence to Islamic dietary guidelines or the use of culturally familiar imagery, were more favorably received. Scores were categorized as follows: 0%-39% (not suitable), 40%-69% (adequate), and 70%-100% (superior).

A study assessing SAM’s interrater reliability for written stroke education materials showed that most individual SAM items had high interrater reliability, with 17 out of 22 items achieving substantial, almost perfect, or perfect weighted κ values (≥0.60), with a total agreement of 96% [15].

### Data Analysis

Data analysis was conducted based on the extracted data from the included apps. The extracted data were first tabulated across all studies, and then the collected data were analyzed using IBM SPSS Statistics (version 25.0). Descriptive statistics, including mean and SD, were calculated for the app ratings from the MARS, Coverage and Depth of Information Checklist, and SAM. This analysis provided a comprehensive overview of app quality, content coverage, and suitability. The research team adhered to ethical principles, including honesty and trustworthiness, in data analysis and when presenting the study’s findings. To protect the rights of the app developers, the names of the apps were identified by codes in this systematic evaluation.

### Ethical Considerations

The research study was approved by the Research Ethics Committees of Mashhad University of Medical Sciences, Mashhad, Iran (IR.MUMS.REC.1400.179). Since the study involved the assessment of publicly available MHAs, no personal data or identifiable participant information were collected. The apps evaluated are commercially available and publicly accessible, ensuring user privacy and confidentiality. No compensation was provided or required for this study as it involved the assessment of publicly available MHAs and not the participation of individuals.

## Results

### App Selection Process

**Figure 1:**
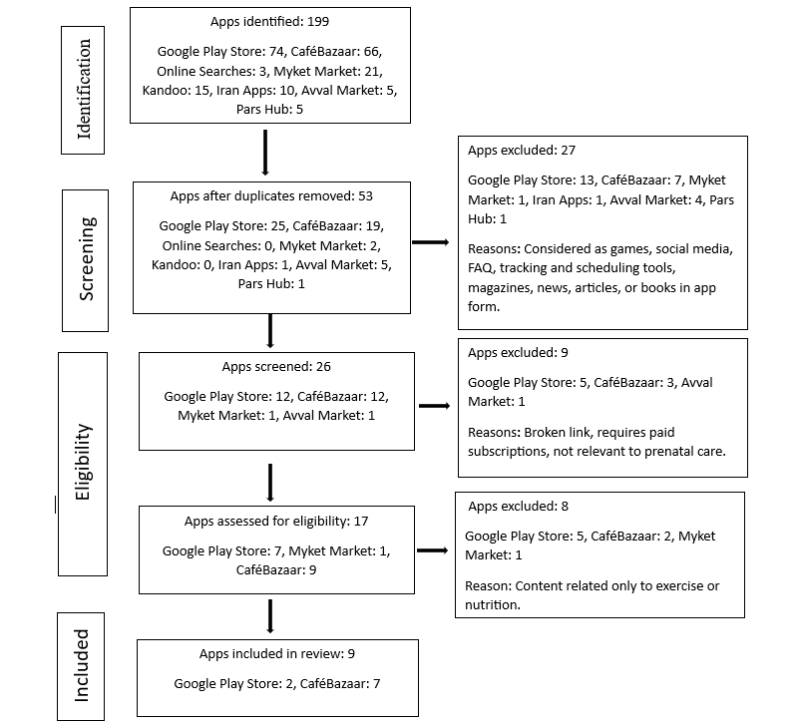
Flow diagram for apps' selection.

The app selection process for this study is outlined in Figure 1. A comprehensive search identified 199 pregnancy-related apps from multiple sources, including internet-based databases, major app stores, and local markets, using the keywords “Pregnancy,” “Pregnant,” “Pregnancy care,” and “Prenatal care.” During the initial screening, 146 duplicate entries were removed. For apps found on multiple platforms, the version from the platform with the higher download count was retained for evaluation, ensuring only one version of each app was included. This process left 53 unique apps for further evaluation. These apps were screened for relevance to pregnancy and availability in the Persian language. During this screening process, 27 apps were excluded because they either did not focus on pregnancy or were not available in Persian, leaving 26 apps for further review. The remaining 26 apps were then assessed against the study’s predefined inclusion criteria, which considered factors such as app functionality, comprehensiveness of content, and language suitability. In this phase, 9 apps were excluded for failing to meet these criteria. This left 17 apps for a detailed eligibility assessment, during which 8 additional apps were excluded for not fully aligning with the requirements. Ultimately, 9 apps (2 apps were available on the Google Play Store, and 7 apps were available on CaféBazaar) met all criteria and were selected for a comprehensive evaluation regarding their quality, content, and suitability for Persian-speaking pregnant women.

### Description of the Selected Apps

Of the 9 apps reviewed, 2 were sourced from Google Play and 7 from CaféBazaar. All selected apps were freely available for download. These apps were commercially developed and lacked affiliations with government agencies, academic institutions, or clinical trials. None had associated scientific publications.

A total of 2 apps had substantial user bases with more than 500,000 downloads each, while 3 apps had more modest download numbers of over 10,000. User ratings ranged from 2.9 to 4.7 stars, with most apps (7 out of 9) receiving ratings of 4.2 or higher. We considered the star ratings that appeared on the platform where the app had the highest number of downloads. In these platforms, the star rating system ranges from 1 star (the lowest rating) to 5 stars (the highest rating).

In total, 5 apps required in-app purchases for full functionality. Notable features across the apps included personal profile creation (6 apps), multilingual support (1 app), and offline functionality (2 apps). Only 2 apps provided transparency about their development and scientific teams. Table 1 summarizes these characteristics. Multimedia Appendix 3 provides the original ratings for all included apps.

**Table 1:** Summary of app characteristics.

App Code^a^	Downloads	Star rating	In-app purchases	Offline functionality	Language	User interaction	Scientific team
APP N1	>500,000	4.5	Yes	No	Persian	Yes	Yes
APP N2	>500,000	4.7	Yes	No	Persian	Yes	No
APP N3	>10,000	4.2	No	Yes	Persian	No	No
APP N4	>10,000	4.3	Yes	No	Persian	Yes	No
APP N5	>10,000	4.7	Yes	No	Persian	Yes	No
APP N6	>50,000	4.7	No	Yes	Persian	No	No
APP N7	>100,000	2.9	No	No	Persian	Yes	No
APP N8	>100,000	4.5	Yes	No	Persian	Yes	Yes
APP N9	>50,000	4.5	No	No	Persian or English	Yes	No

^a^APP N refers to the app code number (eg, APP N1 refers to app code number 1).

### App Quality Assessment

The MARS tool evaluation revealed varying quality levels across the 9 apps. The scores ranged from 2.1 to 3.75 out of 5, with most apps (7 out of 9) scoring above 3. Reviewer-specific scores are presented in Multimedia Appendix 3.

Looking at the subscales, Aesthetics and Functionality emerged as the strongest domains, with median scores of 4. Engagement showed moderate performance, with a median score of 3, while Information quality was generally lower, with a median score of 2.8.

APP N4 demonstrated the strongest performance in three domains (Engagement: 4.2, Functionality: 5, Aesthetics: 4.6), while APP N8 led in Information quality (3.5). The lowest-performing app across most domains was APP N3, as shown in Table 2.

Relationship between app features examining the data qualitatively reveals several patterns. First, apps with higher MARS scores (above 3.5) generally received better user ratings, with most having ratings of 4.5 stars or higher. However, this trend was not consistent for all apps. For instance, APP N2 received high user ratings (4.7 stars) despite having a relatively low score in the information quality domain (2.8).

Second, the two most downloaded apps, with more than 500,000 downloads each, shared specific features. These included in-app purchases, user interaction capabilities, and regular content updates. Nevertheless, a higher number of downloads did not necessarily correlate with higher MARS scores, suggesting that download numbers alone are not a reliable indicator of app quality.

Finally, apps developed by teams with scientific expertise (2 out of 9) tended to score higher in the information quality domain, with scores exceeding 3.2. Conversely, apps with offline functionality (2 out of 9) tended to have lower overall MARS scores compared with apps with internet-based functionality, suggesting that offline accessibility may be associated with compromises in other quality domains such as engagement or information quality.

**Table 2:** MARS scores for antenatal apps.

App code^a^	Overall MARS score	Engagement	Functionality	Aesthetics	Information
APP N4	3.75	4.2	5	4.6	2.2
APP N2	3.62	3.6	4.1	4	2.8
APP N9	3.62	3.8	4.1	4.1	2.8
APP N1	3.57	3.4	3.8	3.9	3.2
APP N8	3.55	3.6	3.8	4	3.5
APP N5	3.35	3.5	4.2	3.9	1.8
APP N7	3.1	2.9	2.5	3.2	3.5
APP N6	2.27	1.7	2.5	2	2.6
APP N3	2.1	1.6	3	1.3	2

^a^APP N refers to the app code number (eg, APP N1 refers to app code number 1).

### Coverage and Depth of Information

Most apps provided relatively poor coverage and depth of health information. Table 3 summarizes the completeness of the content.

None of the apps addressed all the essential training subjects recommended by the Deputy Minister of Health. A total of 6 apps were rated as poor for coverage and depth of health information and 3 apps were rated as adequate. Topics such as sexual health, oral health, immunization, substance avoidance, stress management, and prenatal classes were either poorly covered or entirely neglected. Furthermore, none of the apps provided references for the educational content, raising concerns about the accuracy and reliability of the information.

**Table 3:** Coverage and depth of information in apps.

App code^a^	Overall coverage rating	Key topics covered	Missing topics
APP N1	Adequate	Pregnancy changes, fetal growth, physical activity	Sexual health, immunization, stress management
APP N2	Adequate	Nutrition, common complaints, warning signs	Oral health, prenatal classes, substance use
APP N3	Poor	Pregnancy changes, physical activity	Comprehensive information on health subtopics
APP N4	Adequate	Fetal growth, physical activity, nutrition	Sexual health, immunization, substance use
APP N5	Poor	Common complaints, warning signs	Oral health, prenatal classes, stress management
APP N6	Poor	Basic pregnancy changes	Comprehensive coverage on health subtopics
APP N7	Poor	Common complaints	Sexual health, immunization, substance use
APP N8	Adequate	Physical activity, nutrition, common complaints	Sexual health, prenatal classes, stress management
APP N9	Adequate	Pregnancy changes, fetal growth, physical activity	Oral health, immunization, substance use

^a^APP N refers to the app code number (eg, APP N1 refers to app code number 1).

### Suitability of Information

The SAM assessment revealed varying levels of content suitability across apps. A total of 4 apps achieved superior ratings (>70%), while 5 were rated as adequate (40%-70%) as shown in Table 4.

In terms of Literacy and Layout, high-performing apps (SAM>70%) consistently showed strong literacy demand scores, with layout quality typically aligning with overall suitability ratings. Learning stimulation emerged as the most challenging domain, being consistently the lowest-scoring area across all apps. Even apps with superior overall performance demonstrated significant room for improvement in user engagement strategies.

Cultural appropriateness presented a particularly interesting dimension, with scores ranging widely from 35% to 95%. Notably, apps with superior overall ratings typically exhibited more refined cultural adaptation, suggesting a strong correlation between cultural sensitivity and overall app quality. Apps with culturally misaligned content, such as multimedia depicting non-Iranian contexts or dietary advice unsuitable for local practices, were noted as less effective in meeting user needs. Conversely, apps incorporating culturally aligned recommendations, such as content adhering to Islamic dietary laws or featuring culturally relevant imagery, were better received.

Diving deeper into the quality patterns, apps with superior SAM ratings (>70%) shared several common characteristics. These apps distinguished themselves through comprehensive content organization, clear visual hierarchies, consistent cultural adaptation, and robust interactive elements. This suggests that successful pregnancy apps go beyond mere information delivery, focusing on user experience and cultural relevance.

Conversely, most apps revealed consistent areas requiring improvement. These included enhancing learning stimulation features, developing original educational media, ensuring cultural consistency, and providing authoritative reference citations. These gaps highlight the potential for future app development in the pregnancy support digital ecosystem, pointing to opportunities for creating more engaging, culturally sensitive, and scientifically grounded mHealth resources.

**Table 4:** SAM scores for suitability of app information.

App code^a^	Total score (%)	Literacy demand (%)	Layout and type (%)	Learning stimulation and motivation (%)	Cultural appropriateness (%)
APP N1	81.25% (Superior)	90% (Superior)	85% (Superior)	55% (Adequate)	95% (Superior)
APP N2	75% (Superior)	88% (Superior)	82% (Superior)	50% (Adequate)	80% (Superior)
APP N3	42% (Adequate)	48% (Adequate)	30% (Not suitable)	30% (Not suitable)	60% (Adequate)
APP N4	84.25% (Superior)	92% (Superior)	90% (Superior)	75% (Superior)	80% (Superior)
APP N5	70% (Superior)	85% (Superior)	70% (Superior)	65% (Adequate)	60% (Adequate)
APP N6	44 % (Adequate)	40% (Adequate)	35% (Not suitable)	25% (Not suitable)	35% (Not suitable)
APP N7	48.75 % (Adequate)	75% (Superior)	50% (Adequate)	30% (Not suitable)	40% (Adequate)
APP N8	67.5 % (Adequate)	75% (Superior)	80% (Superior)	50% (Adequate)	65% (Adequate)
APP N9	68.75 % (Adequate)	80% (Superior)	75% (Superior)	55% (Adequate)	65% (Adequate)

^a^APP N refers to the app code number (eg, APP N1 refers to app code number 1).

## Discussion

### Principal Findings

This study evaluated the quality, content accuracy, and user suitability of 9 popular Persian MHAs designed for prenatal care, using standardized assessment tools. The findings revealed that while the apps generally performed well in aesthetics and functionality, they showed notable deficiencies in information quality and coverage. Only a third of the apps achieved adequate health information standards, and none excelled in this category. Despite moderate user ratings, the results highlight significant gaps in the educational and informational content of these apps, underscoring the need for improved standards in app development to better serve expectant mothers.

#### App Availability and Characteristics

Based on the results, no apps were found with any background of scientific documents or being based on the evidence, including the results of the clinical trials. All reviewed apps lacked transparency regarding affiliations and were set up to be commercial rather than as an intervention to change health behavior. All included apps were mostly commercial and were not designed by university academics or research staff. In line with the results of our study, Musgrave et al [16] also indicated that, in their review study of pregnancy apps available in Australia, the affiliations and sources of funding information indicated that all apps were commercially developed and the scientific reviewer teams were not introduced. As a result, this is one of the weaknesses of apps because in order to increase their reliability, the scientific staff or resources for training must be specified [17]. This finding is particularly concerning given the critical role of accurate and comprehensive health information in antenatal care. The qualitative study on mothers’ views on mHealth in self-care for pregnancy identified the need for reliable and trustworthy information in pregnancy apps [12]. Pregnant women were found to be interested in using apps for self-care, but they required reliable and accurate information to make informed decisions about their health [12]. Research highlights the importance of reliable content in health apps. They report that only 5% of the examined apps used reliable information resources, which is a significant concern given the importance of accurate information for pregnant women [11].

In our study, of the 6 apps scoring highest for quality, only 2, APP N7 and APP N9, did not contain in-app purchases. This finding aligns with another review article investigating nutrition-based pregnancy apps, which reported that highly rated MARS apps often required in-app purchases and could not be operated without internet access [18]. Concurrently, recent data suggest that only 5%-10% of app users are willing to pay for in-app purchases [19]. Furthermore, in our study, most applications could not be used without internet access, with only 2 apps offering plain textual information available offline. Similarly, the study by Musgrave et al [16] identified the lack of access to app content without an internet connection as a limitation of mHealth.

#### App Quality Assessment

The MARS tool revealed a nuanced quality landscape. With an average score of 3.55 out of 5, the apps demonstrated moderate quality. Aesthetics and Functionality emerged as the strongest domains, while Information quality consistently scored lower. The high user ratings and significant download numbers of apps such as APP N4 and APP N1 reflect their popularity and perceived utility among users. These apps scored well in terms of user engagement, functionality, and aesthetics, as evidenced by their high MARS scores. This aligns with the general trend observed in health app evaluations where engaging, visually appealing, and easy-to-use apps tend to garner higher user satisfaction [20]. For instance, APP N4 excelled in the engagement and functionality domains with scores of 4.2 and 5, respectively. This suggests that users value interactive and well-designed interfaces that enhance their overall experience. This finding is consistent with studies noting that apps with high engagement features often receive favorable user feedback and higher ratings, even if their informational content is not comprehensive [21].

However, despite their high user ratings, these apps often fall short of delivering thorough educational content. This discrepancy between user satisfaction and content quality highlights a critical issue in the design and development of health apps [22]. Users may prioritize user experience and accessibility over the depth and accuracy of information, which can lead to gaps in the provision of comprehensive health education [23].

#### Content Comprehensiveness

Our study found significant deficiencies in the coverage and depth of information provided by most apps. Despite their high engagement scores, many apps scored poorly in the information domain of MARS and lacked comprehensive coverage of essential pregnancy-related topics. For example, none of the apps covered all the crucial educational topics outlined by the Deputy Minister of Health, and several key areas, such as sexual health and prenatal classes, were consistently neglected. Also, the lack of verifiable sources for the educational content in the reviewed apps further exacerbates this issue, as it raises questions about the accuracy and credibility of the information disseminated to users.

While some apps offered a higher percentage of educational content coverage needed for pregnancy, they were inadequate or neglectful in more than half of the apps on topics such as sexual health, immunization, stress management, and introducing prenatal classes. This observation aligns with findings from another review, which reported that only 16 (31.4%) apps contained information on appropriate pregnancy weight gain as defined by the Institute of Medicine guidelines [18].In addition, a previous study by Tinius et al [24] on apps related to physical activity during pregnancy found that none of the included apps incorporated goal-setting in alignment with the American College of Sports Medicine (ACSM) and American College of Obstetricians and Gynecologists (ACOG) guidelines. The most frequently covered topics in the apps were changes during pregnancy, fetal growth, physical activity, nutrition, common complaints, and warning signs, consistent with other reviews [25]. Overall, reviewers noted that nearly half of all apps were poor or inadequate for recommending to others.

#### Suitability and Cultural Relevance

The evaluation of the apps using the SAM tool revealed mixed results regarding the suitability of health information. While 4 out of 9 apps (APP N1, APP N2, APP N4, and APP N5) were rated as superior in terms of suitability, the majority were merely adequate, and none were found to be unsuitable. This suggests that while some apps do meet the basic requirements for suitable health information materials, there is substantial room for improvement.

Notably, APP N1 and APP N4 received the highest scores in the SAM evaluation, reflecting their superior suitability for the intended audience. These apps likely benefited from their engaging and user-friendly design, which aligns with findings from previous studies indicating that well-designed health information materials are more likely to be effective [12].

Although many apps were evaluated as good to excellent on the SAM score across dimensions such as literacy demand, layout, and type, they received lower scores in the areas of graphics and illustrations and learning stimulation motivation. Similarly, a review of apps for infant feeding reported that 42% of the apps were rated as superior, 54% as adequate, and 3% as unsuitable [26]. However, Cheng et al [26] noted lower scores for readability and cultural appropriateness, which contrasts with our results. Our study determined that most programs were culturally appropriate for Iranian users. This discrepancy may be attributed to the fact that many of the apps in the study by Cheng et al [26] were developed outside of Australia, specifically in America, the United Kingdom, and the European Union, where cultural differences are expected. In contrast, the apps examined in our study were all designed by Iranian teams, which likely contributed to their relative cultural appropriateness.

A unique strength was the cultural appropriateness of these Iranian-developed apps. Unlike international apps that may struggle with cultural adaptation, these apps demonstrated a strong understanding of local user needs. However, areas for improvement included original educational media and interactive elements. These insights underscore the need for app developers to focus on comprehensive design and cultural considerations to enhance both the quality and user experience of pregnancy apps [27].

The study reveals a critical gap between app popularity and quality. High download numbers and user ratings do not guarantee comprehensive or reliable health information. This underscores the urgent need for rigorous content development standards, transparent scientific affiliations, comprehensive educational coverage, and enhanced user engagement strategies.

### Strengths and Limitations

The study had several strengths. It used a comprehensive evaluation framework using 2 independent reviewers, which enhanced the reliability of the assessments. Concentrating on Persian MHAs for prenatal care, the study filled a significant gap in the literature and highlighted areas for improvement, particularly regarding the coverage and depth of health information. However, our study had several limitations. The evaluation was restricted to apps available on Android Play stores available in Iran, potentially excluding other resources on different platforms or less accessible databases. In addition, a key limitation is the rapidly evolving MHA market. Some apps included in this review may no longer be available, and new apps may have emerged since the data collection, which could affect the relevance of our findings. Our study also did not explore the long-term user engagement or the impact of these apps on user health outcomes, which could provide deeper insights into their effectiveness.

### Implications of Findings

The implications of these findings are significant for both app developers and health care providers. High user engagement and aesthetic appeal are crucial for attracting users, but the ultimate value of antenatal apps lies in their ability to deliver reliable, comprehensive health education. Given the increasing reliance on digital tools for health information, ensuring that apps provide accurate, well-rounded educational content is essential. Our study underscores the need for more rigorous standards and oversight in the development and evaluation of health apps to ensure they meet the informational and usability needs of their users. For health care providers, these results suggest caution when recommending apps to expectant mothers. Providers should consider not only the popularity and user ratings of an app but also its content quality and the credibility of the information it provides.

### Future Research Directions

Future research should expand the scope to include a wider range of platforms and perhaps a broader geographic scope to capture a more comprehensive view of available antenatal apps. Further studies should also look into the longitudinal impact of these apps on maternal health outcomes and user behavior. In addition, exploring user feedback and integrating it into apps’ evaluation could provide a more nuanced understanding of app performance and areas for improvement. Given the rapid evolution of digital health tools, continuous monitoring and evaluation are necessary to keep up with emerging trends and ensure that these tools remain relevant and useful for their intended audiences.

### Conclusion

A systematic evaluation of MHAs for prenatal care in Iran revealed a critical need for stricter quality control. While numerous pregnancy apps exist, many lack the quality and comprehensive content mandated by the Ministry of Health. Furthermore, the accuracy of educational content is questionable due to the absence of reliable references or involvement of health care professionals. This research highlights the importance of evaluating app quality and suitability for user navigation while also emphasizing the need to assess the use of behavioral change techniques like goal setting and self-monitoring. By identifying these gaps and deficiencies, researchers can recommend improvements and integrate evidence-based strategies to enhance the effectiveness of pregnancy apps in promoting healthy behaviors and ultimately improving maternal and infant health outcomes.

## Appendix

Multimedia Appendix 1 Clinical Standards and Guidelines by Iran’s Ministry of Health.

Multimedia Appendix 2 Checklist for Evaluating Mobile Health Applications for Pregnant Women.

Multimedia Appendix 3 Original ratings for mobile health app evaluation.

## Abbreviations

ACOG: American College of Obstetricians and Gynecologists
ACSM: American College of Sports Medicine
CFI: comparative fit index
MARS: Mobile App Rating Scale
MHA: mobile health app
mHealth: mobile health
PROSPERO: International Prospective Register of Systematic Reviews
RMSEA: root-mean-square error of approximation
SAM: Suitability Assessment of Materials
SRMR: standardized root-mean-square residual
TLI: Tucker-Lewis index

## Data Availability

All data generated or analyzed during this study are included in this published article and its supplementary information files.
