# Supplementary material for: Evaluating the Quality, Content Accuracy, and User Suitability of mHealth Prenatal Care Apps for Expectant Mothers: Critical Assessment Study
Source: Asian Pac Isl Nurs J. 2025 Feb 13;9:e66852. doi: 10.2196/66852 (PMC11888006; doi:10.2196/66852)
Supplement: Multimedia Appendix 1 [file apinj_v9i1e66852_app1.pdf]

جمهوری اسلامی ایران
وزارت بهداشت، درمان و آموزش پزشکی

معاونت درمان

راهنمای بالینی و استاندارد

خدمات قابل ارائه به زنان و مادران باردار و نوزادان (گروه مامایی)

ماده ۴۵ قانون حمایت از خانواده و جوانی جمعیت

تابستان ۱۴۰۱

تنظیم و تدوین:

سرکار خانم دکتر مرضیه وحید دستجردی دبیر بورد رشته تخصصی زنان و زایمان

سرکار خانم دکتر فاطمه ناهیدی دبیر بورد مامایی

سرکار خانم دکتر فرح بابایی رییس اداره مامایی مرکز مدیریت بیمارستانی و تعالی خدمات بالینی

سرکار خانم دکتر نسرین چنگیزی رییس اداره سلامت مادران دفتر سلامت جمعیت، خانواده و مدارس

سرکارخانم دکتر شهلا خسروی مشاور وزیر در امور مامایی

سرکار خانم دکتر ناهید خدا کرمی رییس انجمن علمی مامایی ایران

سرکار خانم دکتر ملیحه کیان فر رییس اداره سلامت میانسالان

سرکار خانم دکتر پروین عابدی عضو محترم هیات علمی دانشگاه علوم پزشکی اهواز

سرکار خانم فریبا عباسی خامنه کارشناس اداره مامایی مرکز مدیریت بیمارستانی و تعالی خدمات بالینی

سرکار خانم دکتر لیلا اله قلی کارشناس اداره مامایی مرکز مدیریت بیمارستانی و تعالی خدمات بالینی

سرکار خانم زهره مظاهری پور کارشناس اداره مامایی مرکز مدیریت بیمارستانی و تعالی خدمات بالینی

سرکار خانم لاله رادپویان کارشناس اداره سلامت مادران دفتر سلامت جمعیت، خانواده و مدارس

سرکار خانم لیلا هادی پور جهرمی کارشناس اداره سلامت مادران دفتر سلامت جمعیت، خانواده و مدارس

سرکار خانم مینا طباطبایی کارشناس اداره سلامت میانسالان دفتر سلامت جمعیت، خانواده و مدارس

سرکار خانم دکتر ساناز بخشنده رییس گروه تدوین استاندارد و راهنمای بالینی

سرکار خانم آزاده حقیقی کارشناس معاونت درمان

سرکار خانم مرجان مستشار نظامی کارشناس معاونت درمان

با همکاری:

مرکز مدیریت بیمارستانی و تعالی خدمات بالینی

اداره سلامت مادران دفتر سلامت جمعیت، خانواده و مدارس

تحت نظارت فنی:

دکتر سید موسی طباطبایی لطفی

دکتر ساناز بخشنده

گروه تدوین استاندارد و راهنماهای سلامت

دفتر ارزیابی فن آوری، تدوین استاندارد و تعرفه سلامت

مقدمه:

این راهنما بر اساس ماده ٤٥ قانون حمایت از خانواده و جوانی جمعیت با شرح : "شورای عالی بیمه مکلف است راهنمای بالینی استاندارد پوشش بیمه ای خدمات سلامت زنان، مادران باردار و نوزادان را از جمله ماماها و پزشکان در مراکز خصوصی و دولتی در قالب سطح بندی خدمات با لحاظ نظام ارجاع تدوین نماید " می باشد.

انجام مراقبت های مستمر مامایی شامل مراقبت های پیش از بارداری، دوران بارداری و پس از زایمان هنگام ویزیت نقش بسیار مهمی در تشخیص زودرس و درمان به موقع و موثر عوارض بارداری و پس از زایمان دارد. در این مراقبت ها با بررسی وضعیت سلامت مادر و جنین، تعیین سن حاملگی و انجام معاینات بالینی و آزمایشات لازم و تشخیص زود هنگام نشانه‌های خطر و ...، مسائلی مثل فشارخون بالا، کم خونی، دیابت، بیماری‌های عفونی، ناسازگاری‌های خونی بین مادر و جنین، وزن‌گیری نامناسب و اختلالات شایع روانی دوران بارداری و پس از آن... کشف می‌شود که برخورد درست با هریک از این موارد منجر به یک بارداری با حداقل عوارض می‌گردد.

از آن جایی که کلیه مراقبت های مستمر مامایی که به لحاظ ماهیتی جزء خدمات سطح اول محسوب می شوند در قالب ویزیت صورت می گیرد و با توجه به این که اثر بخشی این خدمات در گرو ارائه آن به صورت ادغام یافته ، مستمر و پیوسته می باشد لذا خدمات قابل ارائه به زنان و مادران باردار و نوزادان (گروه مامایی) به تبعیت از آیین نامه ها ، راهنماهای بالینی و دستورالعمل های ابلاغی وزارت متبوع در دوره های پیش از بارداری ، بارداری و پس از زایمان و سلامت زنان در سنین مختلف تدوین شده است.

الف) عنوان دقیق خدمت مورد بررسی (فارسی و لاتین) به همراه کد ملی:

ردیف	کد ملی (RVU)	شرح کد(خدمت)
۱	۹۰۳۰۰۰	برگزاری کلاس آمادگی برای زایمان از هفته ۲۰ تا ۳۷ بارداری به ازای هر جلسه فردی ۹۰ دقیقه
۲	۹۰۳۰۰۵	برگزاری کلاس آمادگی برای زایمان از هفته ۲۰ تا ۳۷ بارداری به ازای هر جلسه گروهی ۹۰ دقیقه به ازای هر بیمار(حداقل ۵ و حداکثر ۱۰ نفر)
۳	۵۰۱۸٦۰	کارگذاری وسیله داخل رحمی (مثل IUD)*
٤	۵۰۱۸٦۵	خارج کردن وسیله داخل رحمی (مثل IUD)
۵	۵۰۲۰۹۰	آزمون بدون استرس جنین (NST)
٦	۵۰۱۷۹۲	نمونه‌برداری اندوسرویکال (پاپ اسمیر) (عمل مستقل)

* مطابق با نامه شماره ٦٩٢٧٣ مورخ ١٤٠١/٠٤/٢٨ دبیر محترم ستاد ملی جمعیت، بر اساس تبصره ماده ۵۰ قانون حمایت از خانواده و جوانی جمعیت، هرگونه ارایه داروهای جلوگیری از بارداری در داروخانه های سراسر کشور و شبکه بهداشت و کار گذاشتن اقلام پیشگیری، صرفا باید با تجویز پزشک باشد و ماماها نمی توانند بدون نسخه پزشک اقدام به کارگذاری IUD نمایند.

ب) تعریف و تشریح خدمت مورد بررسی :

خدمات قابل ارائه به زنان و مادران باردار و نوزادان که توسط ماما ارائه می شود شامل آموزش، مشاوره ، غربالگری (به جز بررسی ناهنجاری ژنتیکی جنین) مراقبت های پیش از بارداری، بارداری و پس از زایمان، و بررسی سلامت زنان در سنین مختلف، تجویز دارو و درخواست خدمات پاراکلینیک (آزمایشات و تصویربرداری) می باشد.

ج) اقدامات یا پروسیجرهای ضروری جهت درمان بیماری:

- **ارزیابی قبل از انجام پروسیجر**

- بررسی وضعیت بارداری فعلی، سابقه بارداری وزایمان قبلی، سابقه بیماری ها ،ارزیابی الگوی تغذیه ، غربالگری سلامت روان، بررسی رفتارهای پر خطرو اعتیاد ، مصرف سیگار / الکل ، مصرف مکمل های غذایی.
- بررسی تاریخچه باروری و سلامت زنان عفونت های جنسی
- ارزیابی اولیه نوزاد در بیمارستان

- **ارزیابی حین انجام پروسیجر**

عنوان خدمت قابل ارائه	آموزش ، مشاوره و ارزیابی سلامت زنان در سنین مختلف	پیش از بارداری	حین بارداری	پس از زایمان
**معاینات بالینی**	اخذ شرح حال و تاریخچه سلامت کلی زنان در سنین مختلف<br>معاینه ژنیکولوژی از نظر وجود زخم تناسلی/نمای غیر طبیعی سرویکس، بررسی عفونت های جنسی، بررسی ضایعات خونریزی دهنده ، بررسی مشکلات اوروژنیتال یائسگی<br>بررسی علائم و نشانه برای تشخیص زود هنگام سرطان دهانه رحم و غربالگری سرطان دهانه رحم (تهیه نمونه اچ پی وی و پاپ اسمیر)<br>بررسی بیماری های پستان، بررسی عوامل خطر و غربالگری و تشخیص زود هنگام سرطان پستان<br>غربالگری سلامت روان	گرفتن شرح حال و معاینه فیزیکی دهان و دندان،پوست، پستان، شکم، اندام، واژن و لگن<br>غربالگری سلامت روان	اندازه گیری قد و وزن، تعیین نمایه توده بدنی، ترسیم جدول وزن گیری، علائم حیاتی، معاینه فیزیکی، کنترل صدای قلب جنین، اندازه گیری ارتفاع رحم، معاینه دهان و دندان، معاینه شکم(مانور لئوپولد) ، غربالگری سلامت روان	انجام معاینه بالینی (علائم حیاتی، معاینه فیزیکی)<br>غربالگری سلامت روان (جهت تشخیص اختلالات شایع روانی post partum)
**درخواست آزمایشات**	تشخیص حاملگی (خون و ادرار)<br>تهیه نمونه HPV- پاپ اسمیر و درخواست بررسی HPV- نمونه پاپ اسمیر<br>Rapid Test HIV<br>FBS,Cholestrol	CBC,TSH,FBSو HBsAg، پاپ اسمیر(کد ملی : ۵۰۱۷۹۲) و تیتر آنتی بادی ضد سرخجه	CBCdiff, BG, Rh ,U/A, U/C,BUN, Creatinine,FBS,BS,GTT, GCT ,HBsAg، VDRL ،HIV،HIVAb, ، کومبس غیر مستقیم ، TSH	انجام پاپ اسمیر(کد ملی : ۵۰۱۷۹۲)،<br>در مبتلایان به دیابت بارداری:درخواست FBS,OGTT(ناشتا و

	کامل ادرار و کشت ادرار<br>کشت ترشحات واژن، Prolactin, ,<br>LH ,FSH , SGOT, SGPT	HIV، وHIVAb,<br>VDRL(دررفتارهای پرخطر) **-HPVپاپ اسمیر**<br>کشت ترشحات واژن،		دوساعته)
**درخواست رادیولوژی و سونوگرافی**	ماموگرافی<br>سونوگرافی پستان<br>سونوگرافی رحم و ضمائم	سونوگرافی برای بررسی وضعیت رحم و ضمائم	سونوگرافی بارداری روتین ۷۰۱۷۱۵) در هفته ٦ تا ۱۰ در هفته ۱٦ تا ۱۸ بارداری و هفته ۳۱تا ۳٤ بارداری به منظور بررسی وضعیت جنین، محل جفت	
**تجویز دارو و مکمل ها**	علائم یائسگی<br>بیماری های پستان مانند ماستیت	اسید فولیک	اسید فولیک از ابتدای بارداری تا پایان بارداری<br>آهن و مولتی ویتامین مینرال از شروع هفته ۱٦ بارداری تا پایان بارداری<br>تجویز ویتامین دی از ابتدای بارداری تا پایان بارداری	آهن و مولتی ویتامین مینرال تا ۳ ماه پس از زایمان
**آموزش و مشاوره**	آموزش و بررسی علائم و عوارض یائسگی(خونریزی غیرطبیعی، اختلال وازوموتور، اختلال روابط زناشویی، و اختلالات دوره یائسگی و ...)<br>آموزش ، مشاوره و بررسی رفتارهای پرخطر و عفونت<br>آموزش ورزش های کگل	فرزندآوری، پیشگیری از سقط ، بهداشت فردی، روان، جنسی، دهان و دندان، تغذیه/ مکمل های دارویی، سو مصرف مواد و الکل، قطع روش پیشگیری از	پیشگیری از سقط، بهداشت فردی و روان و جنسی، بهداشت دهان و دندان، سو مصرف دارو و مواد مخدر، عدم کشیدن سیگار و دخانیات، عدم مصرف الکل، تغذیه و مکمل‌های دارویی، شکایت	بهداشت فردی و روان و جنسی، بهداشت دهان و دندان، تغذیه و مکمل های دارویی، شکایات شایع پس از زایمان، نحوه شیردهی و تداوم آن ، علائم خطر مادر و نوزاد، مراقبت از نوزاد، مشاوره فرزند

	آموزش خود آزمایی پستان، معاینه پستان<br>بررسی از نظر رضایت روابط زناشویی و انجام آموزش، مشاوره و ارجاعات ضروری	بارداری و زمان مناسب باردار شدن، غربالگری و مراقبت زوجین در معرض خطر ناباروری، غربالگری و مراقبت سلامت زناشویی زوجین	شایع بارداری، علائم خطر حین بارداری، مشاوره باروری سالم / شیردهی نوزاد/ فواید زایمان طبیعی، معرفی مادر به کلاس‌های آمادگی برای زایمان (کدهای ۹۰۳۰۰۵ و ۹۰۳۰۰۰)<br>مراقبت از نوزاد، علائم خطر نوزاد، غربالگری کم‌کاری مادرزادی تیروئید و انجام آن در روزهای ۳ تا ۵ تولد،	آوری
**انجام ایمن سازی**		تزریق واکسن توأم (کزاز و دیفتری) و سرخجه **(MMR) و واکسن هپاتیت B**	توام (کزاز و دیفتری) و آنفولانزا (در صورت نیاز) ، ایمونوگلوبولین انتی D، از هفته ۲۸ تا ۳٤ بارداری (در صورت نیاز)	ایمونوگلوبولین انتی D،در مادر ارهاش منفی با نوزاد ارهاش مثبت طی ۷۲ ساعت اول پس از زایمان
**اقدامات تکمیلی بر حسب شرایط**		۱–ارائه اقدامات اولیه مادران عارضه دار (بر اساس علائم و نتایج ارزیابی یا شکایت مستقیم خود مادر) طبق دستورالعمل های کشوری و شکایات شایع<br>۲– ارائه مراقبت از زنان باردار و نوزادان با شرایط خاص اجتماعی<br>۳– شناسایی خانم های نیازمند خدمات تخصصی و ارجاع به متخصص و اعزام مادران نیازمند مراقبت ویژه به بیمارستان<br>– تجویز انواع ویتامین ها به صورت کپسول، قرص، شربت، قطره، پماد (تنها می توان ویتامین های گروه B را به صورت آمپول تجویز نمود)<br>–ترکیبات آهن و مواد معدنی به صورت کپسول، قرص، شربت، قطره خوراکی<br>–داروهای ضد درد شامل : هیوسین، استامینوفن، مفنامیک اسید، دیکلوفناک ، پیروکسیکام، ایبوبروفن، ژلوفن، ناپروکسن، ایندومتاسین به صورت قرص، شیاف، آمپول، کپسول، پماد و ژل (فرم تزریقی دیکلوفناک و پیروکسیکام در بیماران غیر مجاز می باشد)		

- کلیه محلول های ضدعفونی

- محلول های تزریقی قندی ۵٪ ، قندی – نمکی ، رینگر، نرمال سالین

- آنتی اسید و ضد نفخ (آلومینیوم ام جی، آلومینیوم ام جی اس، پانکراتین، دایجستیو، دایمتیکون، سایمتدین و رانی تیدین) به صورت قرص و سوسپانسیون

- انواع داروهای ضد تهوع و ضد حساسیت (پرومتازین، متوکلوپرامید، دیفن هیدرامین، آنتی هیستامین دکونژستان) به صورت قرص، شربت ، قطره و آمپول

- ملین ها شامل: بیزاکودیل، پسیلیوم، سی لاکس، هیدروکسید منیزیم، MOM به صورت قرص ، سوسپانسیون، شربت ، شیاف، پودر و ...

- داروهای بی حسی موضعی نظیر لیدوکائین به صورت پماد، ژل

- ترکیبات پروژسترونی تزریقی و خوراکی

- قرص و آمپول مترژن(داخل عضله)

- کپسول ترانگزامیک اسید

-استامینوفن

- پنی سیلین و مشتقات آن شامل ویال های ۸۰۰۰۰۰ ۳-۳-۶ ، LA۱۲۰۰۰۰۰ ، آمپی سیلین (خوراکی) ، سفالکسین خوراکی، داکسی سایکلین، آموکسی سیلین(خوراکی) سفیکسیم، اریترومایسین، ، کلوگزاسیلین با رعایت ملاحظات تشخیصی مثل حساسیت به پنی سیلین و سابقه آلرژی

- قرص های کوتریموکسازول، نالیدیکسیک اسید و سیپروفلوکساسین منوط به نتیجه کشت ادرار

-کرم واژینال (کلیندامایسین/کلوتریمازول/ نیستاتین/ میکونازول/،تریپل سولفا/ مترونیدازول)

-اندانسترون قرص

-متوکلوپرامید قرص

-**ویتامین ب ٦** خوراکی

-مترونیدازول قرص خوراکی

- داروهای ضدخارش ایمن در حاملگی ، پماد کالامین D ، کالامین ، تریامسینولون NN تریامسینولون، ، هیدروکورتیزون، کالاندولا

- فلوکونازول به صورت قرص و کپسول

- آسیکلوویر به صورت پماد و قرص

- **ارزیابی بعد از انجام پروسیجر:**

تعیین وقت ویزیت بعدی

- **کنترل عوارض جانبی انجام پروسیجر:**

- درصورتی که مادر عارضه دار و نیازمند مراقبت ویژه بوده به متخصص زنان و زایمان ارجاع می شود.

د) تواتر ارائه خدمت (تعداد دفعات مورد نیاز / فواصل انجام):

- پیش از بارداری : حداقل یک بار و با توجه به وضعیت فرد می تواند افزایش یابد
- حین بارداری:حداقل ۸ ویزیت در بارداری معمولی و بدون عارضه و درصورت نیاز با توجه به وضعیت فرد تعداد ویزیت ها افزایش می یابد

ویزیت ۱: هفته ۱۰-٦ یکبار

ویزیت ۲ : هفته ۲۰-١٦ یک بار

ویزیت ۳: هفته ۳۰-٢٤ یک بار

ویزیت ٤: هفته ٣٤-٣١ یک بار

ویزیت ٥: هفته ۳۷-۳٥ یک بار

ویزیت٦و۷و۸ : هفته ٤٠-۳۸ هر هفته یک بار

- پس از زایمان: حداقل ۳ ویزیت و در صورت نیاز افزایش می یابد.

ویزیت ۱: روز ۳-۱ پس از زایمان

ویزیت۲ : روز ۱٥-۱۰ پس از زایمان

ویزیت۳ : روز ٤٢-۳۰ پس از زایمان

ه) افراد صاحب صلاحیت جهت تجویز (Order) / خدمت مربوطه و استاندارد تجویز:

کارشناس مامایی ، کارشناس ارشد مامایی و دکترای (PHD) مامایی، کارشناس ارشد مشاوره مامایی، دکترای بهداشت باروری (PHD)

این افراد باید تحصیلات مامایی را برابر مقررات در مراکز آموزش داخلی و خارجی به پایان رسانیده و موفق به اخد مدرک تحصیلی از مراکز معتبر مورد تایید وزارت بهداشت، درمان و آموزش پزشکی شده باشند.

و) افراد صاحب صلاحیت جهت ارائه خدمت مربوطه:

کارشناس مامایی ، کارشناس ارشد مامایی و دکترای (PHD) مامایی، کارشناس ارشد مشاوره مامایی، دکترای بهداشت باروری (PHD)

این افراد باید تحصیلات مامایی را برابر مقررات در مراکز آموزش داخلی و خارجی به پایان رسانیده و موفق به اخد مدرک تحصیلی از مراکز معتبر مورد تایید وزارت بهداشت، درمان و آموزش پزشکی شده باشند.

ز) عنوان و سطح تخصص های مورد نیاز (استاندارد) برای سایر اعضای تیم ارائه کننده خدمت:

ردیف	عنوان تخصص	تعداد موردنیاز به طور استاندارد به ازای ارائه هر خدمت	میزان تحصیلات حداقل مورد نیاز	سابقه کار و یا دوره آموزشی مصوب در صورت لزوم	نقش در فرایند ارائه خدمت
۱	منشی	یک نفر	دیپلم		پذیرش

ح) استانداردهای فضای فیزیکی و مکان ارائه خدمت:

فضای مورد نیاز برای ارائه خدمت، اتاقی با ابعاد حد اقل 3x4 متر،با تهویه مناسب و نور کافی می باشد.
دفاتر کار مامایی، مراکز مشاوره و ارائه خدمات مامایی، درمانگاههای عمومی و تخصصی ، پایگاه ها و مراکز جامع سلامت، بیمارستان ها، مراکز بهداشتی درمانی

ط) تجهیزات پزشکی سرمایه ای به ازای هر خدمت:

عنوان تجهیزات	تعداد
تخت ژنیکولوژی	یک
تخت معاینه	یک
چراغ پایه دار	یک
فور	یک
پنس جفت	یک
چیتل فورسپس و محل نگهداری آن	یک
گوشی طبی و فشار سنج	یک
پاراوان	یک
ترازوی بزرگسال	یک
قد سنج یا متر نواری	یک
بیکس/دیش درب دار	یک
رسیور	یک
کاسه فلزی	یک
پایه سرم	یک
ترالی	یک
چراغ قوه و باطری	یک
تابوره	یک
سینی استیل	یک
لگن استیل	یک
چهار پایه جلوی تخت	یک

ی) داروها، مواد و لوازم مصرفی پزشکی جهت ارائه هر خدمت:

اقلام مصرفی مورد نیاز	میزان مصرف(تعداد یا نسبت)
ست زایمان	یک
ست آی یو دی	یک
ست پانسمان	یک
اسپکولوم یکبار مصرف	به تعداد لازم
ست پاپ اسمیر	به تعداد لازم
پنبه و موادضدعفونی	به میزان لازم جهت شستشو
دستکش استریل و لاتکس	به تعداد لازم
سرنگ و سر سوزن (در اندازه های مختلف)	به میزان لازم
ماسک	به میزان لازم
تورنیکه	یک
چسب ضد حساسیت	به میزان لازم
آنژیوکت و سه راهی	یک
باند	به میزان لازم
ست سرم	یک
انواع سرم	به میزان لازم
ترمومتر	یک
گان،ملحفه،شان،حوله،رو بالشی	به میزان لازم

ک) استانداردهای ثبت:

گزارش باید شامل مشخصات هویتی دریافت کننده خدمت و مشخصات مرکز ارائه کننده خدمت باشد.

- سوابق پزشکی ، حساسیت های دارویی و غذایی، سوابق بارداری های قبلی در پرونده ثبت شود.
- شکایت اصلی مراجعه کننده و علائم و نشانه ها در پرونده ثبت شود.
- باید نتایج اطلاعات مربوط به معایناتی که انجام می گردد، درگزارش ذکر شود.
- باید نتایج کلیه آزمایشات و سونوگرافی و تصویربرداری های انجام شده ذکر گردد.
- باید گزارش مراقبت ها ، تجویزها وآموزش های انجام شده ثبت گردد.
- در صورتی که خانم نیازمند مراقبت ویژه باشد باید با ذکر دقیق مورد ثبت گردد.
- باید در صورت ارجاع خانم، علت، نوع و نتیجه ارجاع ثبت گردد.

ل) اندیکاسیون های دقیق جهت تجویز خدمت:

- زنان در سنین باروری
- زنانی که قصد بارداری دارند.
- زنان باردار
- زنان زایمان کرده وشیرده
- زنان در دوره یائسگی

م) شواهد علمی در خصوص کنترااندیکاسیون های دقیق خدمت:

کنترااندیکاسیون ندارد.

ن) مدت زمان ارائه هر واحد خدمت:

ردیف	عنوان تخصص	میزان تحصیلات	حداقل مدت زمان مشارکت در فرایند ارائه خدمت	نوع مشارکت در قبل، حین و بعد از ارائه خدمت
۱	ماما / دکتری بهداشت باروری (PHD)	کارشناس/ کارشناس ارشد/ دکترا	۳۰ دقیقه	مصاحبه، معاینه، آموزش و مشاوره و تجویز دارو و درخواست آزمایشات و پاراکلینیک

س) مدت اقامت در بخش های مختلف بستری جهت ارائه هر بار خدمت مربوطه:

خدمت سرپایی بوده و نیاز به بستری شدن ندارد

ع) موارد ضروری جهت آموزش به بیمار:

در ملاقات های مختلف و با توجه به هفته بارداری ، درمورد بهداشت فردی، روانی و جنسی ، بهداشت دهان و دندان ، بهداشت محیط کار، مصرف دارو، منع استعمال دخانیات و کشیدن سیگار ، اهمیت مراقبت های دوران بارداری ، شکایت های شایع و علایم خطر بارداری ، فواید زایمان طبیعی ، شیردهی و شرکت در کلاس های آمادگی برای زایمان به مادر آموزش داده شود.
