# Supplementary material for: Evaluating the Quality, Content Accuracy, and User Suitability of mHealth Prenatal Care Apps for Expectant Mothers: Critical Assessment Study
Source: Asian Pac Isl Nurs J. 2025 Feb 13;9:e66852. doi: 10.2196/66852 (PMC11888006; doi:10.2196/66852)
Supplement: Multimedia Appendix 2 [file apinj_v9i1e66852_app2.docx]

**Checklist for Evaluating Mobile Health Applications for Pregnant Women**

The checklist is structured to evaluate mobile health applications (MHAs) for pregnant women, focusing on the breadth and depth of their content. It ensures alignment with maternal health priorities and app usability standards. The checklist items are grouped into thematic domains and the evaluation system assigns scores to determine the overall performance of the app using a standardized scoring framework.

**Evaluation Framework**

- **Scoring System**:
  - **2 points**: Correct and sufficient information is provided.
  - **1 point**: Partially correct or insufficient information is provided.
  - **0 points**: Information is incorrect, irrelevant, or not addressed.
- **Final Evaluation**:
  - **Superior**: 41–46 points (90%–100%).
  - **Adequate**: 23–40 points (50%–89%).
  - **Poor/Low**: <23 points (≤49%).
- **Instructions for Reviewers**
  - Rate each item using the 0–2 scoring system.
  - Sum scores to determine the total.
  - Categorize app content as Superior, Adequate, or Poor/Low based on the total score.

**Domain 1: Content Relevance and Coverage**

1. **Physiology of Pregnancy**
   - Does the app provide comprehensive and accurate information about pregnancy physiology?
   - Are the stages of pregnancy clearly explained?
2. **Personal Hygiene**
   - Does the app guide maintaining hygiene specific to pregnancy needs?
3. **Sexual Health**
   - Is information on safe sexual practices during pregnancy included?
4. **Oral Health**
   - Does the app address oral hygiene's impact on pregnancy?
5. **Fetal Growth**
   - Are fetal development milestones adequately explained?

**Domain 2: Health Education and Practical Tips**

1. **Physical Activity**
   - Does the app recommend safe exercises and physical activities?
2. **Nutrition**
   - Is there tailored guidance on diet and nutritional requirements?
3. **Taking Pregnancy Supplements**
   - Are recommendations for supplements (e.g., folic acid, iron) provided?
4. **Common Complaints**
   - Does the app offer practical solutions for managing common pregnancy complaints (e.g., nausea, fatigue)?
5. **Warning Signs**
   - Are potential warning signs clearly explained to ensure timely intervention?

**Domain 3: Prenatal Care Guidance**

1. **Prenatal Testing (Maternal Labs)**
   - Is information on recommended laboratory tests provided?
2. **Prenatal Testing (Fetal Screening)**
   - Are fetal screening procedures adequately described?
3. **Immunization**
   - Does the app inform about essential vaccines during pregnancy?
4. **Do Not Take Drugs, Alcohol, Cigarettes**
   - Are there explicit warnings against harmful substances?

**Domain 4: Psychological and Emotional Support**

1. **Stress Management Solutions**
   - Does the app provide tools or techniques for stress reduction?
2. **Preterm Labor Education**
   - Is education on preterm labor signs and prevention included?
3. **Prenatal Classes**
   - Does the app offer information on the availability and content of prenatal classes?
4. **Benefits of Natural and Safe Delivery**
   - Is there an emphasis on the advantages of natural delivery methods?
5. **Partner Education and Support**
   - Does the app include information or resources specifically aimed at educating the partner (husband or significant other) on their role during pregnancy, including emotional support, practical assistance, and participation in prenatal care?

**Domain 5: Late Pregnancy and Labor**

1. **Management of Late Pregnancy Symptoms**
   - Are strategies for managing discomfort in late pregnancy available?
2. **Warning Signs for Pregnancy-Induced Hypertension**
   - Are specific warnings related to hypertension addressed?
3. **Labor and Birth Issues, and When to Call Provider**
   - Does the app guide users on labor onset and when to seek help?

**Domain 6: Postnatal Preparation**

1. **Breastfeeding Training**
   - Are there resources or tips for successful breastfeeding?
