# Supplementary material for: Evaluating the Quality, Content Accuracy, and User Suitability of mHealth Prenatal Care Apps for Expectant Mothers: Critical Assessment Study
Source: Asian Pac Isl Nurs J. 2025 Feb 13;9:e66852. doi: 10.2196/66852 (PMC11888006; doi:10.2196/66852)
Supplement: Multimedia Appendix 3 [file apinj_v9i1e66852_app3.docx]

**Original Ratings for mHealth App Evaluation**

| **App Code** | **APP N1** | | | **APP N2** | | | **APP N3** | | | **APP N4** | | | **APP N5** | | | **APP N6** | | | **APP N7** | | | **APP N8** | | | | **APP N9** | | |
| --- | --- | --- | --- | --- | --- | --- | --- | --- | --- | --- | --- | --- | --- | --- | --- | --- | --- | --- | --- | --- | --- | --- | --- | --- | --- | --- | --- | --- |
| **platform distribution** | CaféBazaar*  Google Play Store | | | Google Play Store*  CaféBazaar | | | CaféBazaar* | | | CaféBazaar*  Kandoo | | | CaféBazaar*  Online Searches  Kandoo | | | Google Play Store*  CaféBazaar | | | CaféBazaar* | | | CaféBazaar*  Google Play Store  Myket Market | | | | CaféBazaar* | | |
| **Most Download Count by Platform** | >500,000 | | | >500,000 | | | >10,000 | | | >10,000 | | | >10,000 | | | >50,000 | | | >100,000 | | | >100,000 | | | | >50,000 | | |
| **Star Rating** | 4.5 | | | 4.7 | | | 4.2 | | | 4.3 | | | 4.7 | | | 4.7 | | | 2.9 | | | 4.5 | | | | 4.5 | | |
| **In-App Purchases** | Yes | | | Yes | | | No | | | Yes | | | Yes | | | No | | | No | | | Yes | | | | No | | |
| **Offline Functionality** | No | | | No | | | Yes | | | No | | | No | | | Yes | | | No | | | No | | | | No | | |
| **Language** | Persian | | | Persian | | | Persian | | | Persian | | | Persian | | | Persian | | | Persian | | | Persian | | | | Persian/English | | |
| **User Interaction** | Yes | | | Yes | | | No | | | Yes | | | Yes | | | No | | | Yes | | | Yes | | | | Yes | | |
| **introduce Scientific Team** | Yes | | | No | | | No | | | No | | | No | | | No | | | No | | | Yes | | | | No | | |
| **Profile Creation Option** | Yes | | | No | | | No | | | No | | | No | | | No | | | No | | | Yes | | | | No | | |
| **Online Services Provided** | Yes | | | No | | | No | | | No | | | No | | | No | | | No | | | Yes | | | | No | | |
| **Multimedia Learning Materials** | Yes | | | No | | | No | | | No | | | No | | | No | | | No | | | Yes | | | | No | | |
| **User Rating** | 3.57 | | | 3.62 | | | 2.1 | | | 3.75 | | | 3.35 | | | 2.27 | | | 3.1 | | | 3.55 | | | | 3.62 | | |
| **Scientific Articles on App Published** | No | | | No | | | No | | | No | | | No | | | No | | | No | | | No | | | | No | | |
| **MARS Scores for App Quality Assessment** | Final score | Rev.1 | Rev.2 | Final score | Rev.1 | Rev.2 | Final score | Rev.1 | Rev.2 | Final score | Rev.1 | Rev.2 | Final score | Rev.1 | Rev.2 | Final score | Rev.1 | Rev.2 | Final score | Rev.1 | Rev.2 | | Final score | Rev.1 | Rev.2 | Final score | Rev.1 | Rev.2 |
| **Engagement** | 3.4 | 3.5 | 3.5 | 3.6 | 3.4 | 3.6 | 1.6 | 1.5 | 1.8 | 4.2 | 4 | 4.2 | 3.5 | 3.5 | 3.5 | 1.7 | 2 | 1.5 | 2.9 | 2.5 | 3 | | 3.6 | 3.5 | 3.8 | 3.8 | 3.8 | 3.5 |
| **Functionality** | 3.8 | 3.75 | 3.8 | 4.1 | 4.5 | 4 | 3 | 3 | 3 | 5 | 5 | 4.8 | 4.2 | 4 | 4.2 | 2.5 | 2.5 | 3 | 2.5 | 3 | 2.5 | | 3.8 | 3.5 | 3.5 | 4.1 | 4.5 | 4 |
| **Aesthetics** | 3.9 | 3.8 | 4 | 4 | 4 | 4 | 1.3 | 1.5 | 1.2 | 4.6 | 4.5 | 4.8 | 3.9 | 3.8 | 4 | 2 | 2 | 2 | 3.2 | 3.5 | 3 | | 4 | 4 | 3.5 | 4.1 | 4 | 4.5 |
| **Information** | 3.2 | 3.5 | 3 | 2.8 | 3 | 2.7 | 2 | 2 | 2.3 | 2.2 | 2 | 2.5 | 1.8 | 2 | 1 | 2.6 | 2.5 | 2.6 | 3.5 | 3.5 | 3.5 | | 3.5 | 3.5 | 3 | 2.8 | 3 | 2.8 |
| **Overall MARS Score** | 3.57 | 3.63 | 3.57 | 3.62 | 3.8 | 3.42 | 2.1 | 2.32 | 1.75 | 3.75 | 4.5 | 3.45 | 3.35 | 3.45 | 3.17 | 2.27 | 2.25 | 2.27 | 3.1 | 3.12 | 3 | | 3.55 | 3.62 | 3.45 | 3.62 | 3.82 | 3.7 |
| **Coverage and Depth of Information** |  | | | | | | | | | | | | | | | | | | | | | | | | | | | |
| **Physiology of Pregnancy** | 2 | | | 2 | | | 1 | | | 2 | | | 2 | | | 1 | | | 1 | | | 2 | | | | 2 | | |
| **Personal Hygiene** | 1 | | | 1 | | | 1 | | | 1 | | | 1 | | | 1 | | | 1 | | | 1 | | | | 1 | | |
| **Sexual Health** | 1 | | | 1 | | | 0 | | | 1 | | | 0 | | | 1 | | | 0 | | | 1 | | | | 1 | | |
| **Oral Health** | 1 | | | 1 | | | 1 | | | 1 | | | 1 | | | 1 | | | 1 | | | 1 | | | | 1 | | |
| **Fetal Growth** | 2 | | | 2 | | | 2 | | | 2 | | | 2 | | | 2 | | | 1 | | | 2 | | | | 2 | | |
| **Physical Activity** | 2 | | | 2 | | | 2 | | | 2 | | | 2 | | | 2 | | | 1 | | | 2 | | | | 2 | | |
| **Nutrition** | 2 | | | 2 | | | 1 | | | 2 | | | 1 | | | 1 | | | 1 | | | 2 | | | | 2 | | |
| **Taking Pregnancy Supplements** | 2 | | | 1 | | | 1 | | | 2 | | | 1 | | | 1 | | | 1 | | | 2 | | | | 2 | | |
| **Common Complaints** | 2 | | | 2 | | | 2 | | | 2 | | | 2 | | | 2 | | | 2 | | | 2 | | | | 2 | | |
| **Warning Signs** | 2 | | | 2 | | | 2 | | | 2 | | | 2 | | | 2 | | | 2 | | | 2 | | | | 2 | | |
| **Prenatal Testing (Maternal Labs)** | 1 | | | 1 | | | 1 | | | 1 | | | 1 | | | 1 | | | 1 | | | 1 | | | | 1 | | |
| **Prenatal Testing (Fetal Screening)** | 1 | | | 1 | | | 1 | | | 1 | | | 1 | | | 1 | | | 1 | | | 1 | | | | 1 | | |
| **Immunization** | 1 | | | 1 | | | 0 | | | 1 | | | 0 | | | 1 | | | 0 | | | 1 | | | | 1 | | |
| **Do Not Take Drugs, Alcohol, Cigarettes** | 2 | | | 2 | | | 2 | | | 2 | | | 2 | | | 2 | | | 1 | | | 2 | | | | 2 | | |
| **Stress Management Solutions** | 1 | | | 1 | | | 1 | | | 1 | | | 1 | | | 1 | | | 1 | | | 1 | | | | 1 | | |
| **Preterm Labor Education** | 1 | | | 1 | | | 1 | | | 1 | | | 1 | | | 1 | | | 1 | | | 1 | | | | 1 | | |
| **Prenatal Classes** | 2 | | | 1 | | | 2 | | | 1 | | | 1 | | | 1 | | | 1 | | | 2 | | | | 1 | | |
| **Benefits of Natural and Safe Delivery** | 1 | | | 1 | | | 1 | | | 1 | | | 1 | | | 1 | | | 1 | | | 1 | | | | 1 | | |
| **Partner Education and Support** | 0 | | | 0 | | | 0 | | | 1 | | | 1 | | | 0 | | | 1 | | | 0 | | | | 1 | | |
| **Management of Late Pregnancy Symptoms** | 1 | | | 1 | | | 1 | | | 1 | | | 1 | | | 2 | | | 1 | | | 1 | | | | 1 | | |
| **Warning Signs for Pregnancy Induced Hypertension** | 1 | | | 1 | | | 2 | | | 1 | | | 1 | | | 1 | | | 1 | | | 1 | | | | 1 | | |
| **Labor and Birth Issues, and When to Call Provider** | 1 | | | 1 | | | 1 | | | 1 | | | 1 | | | 1 | | | 1 | | | 1 | | | | 1 | | |
| **Breastfeeding Training** | 1 | | | 1 | | | 1 | | | 1 | | | 1 | | | 1 | | | 1 | | | 1 | | | | 1 | | |
| **Total Content Coverage Score** | 41 | | | 38 | | | 22 | | | 40 | | | 23 | | | 20 | | | 21 | | | 39 | | | | 38 | | |
| Final Rating | Adequate | | | Adequate | | | Poor | | | Adequate | | | Poor | | | Poor | | | Poor | | | Adequate | | | | Adequate | | |
| **Suitability of Information** |  | | | | | | | | | | | | | | | | | | | | | | | | | | | |
| **Literacy Demand** |  | | | | | | | | | | | | | | | | | | | | | | | | | | | |
| **SAM Content (Purpose)** | 90% (Superior) | | | 90% (Superior) | | | 90% (Superior) | | | 90% (Superior) | | | 90% (Superior) | | | 90% (Superior) | | | 90% (Superior) | | | 90% (Superior) | | | | 90% (Superior) | | |
| **SAM Content (Content Topics)** | 88% (Superior) | | | 88% (Superior) | | | 88% (Superior) | | | 88% (Superior) | | | 88% (Superior) | | | 88% (Superior) | | | 88% (Superior) | | | 88% (Superior) | | | | 88% (Superior) | | |
| **SAM Content (Scope)** | 48% (Adequate) | | | 48% (Adequate) | | | 48% (Adequate) | | | 48% (Adequate) | | | 48% (Adequate) | | | 48% (Adequate) | | | 48% (Adequate) | | | 48% (Adequate) | | | | 48% (Adequate) | | |
| **SAM Content (Summary & Review)** | 92% (Superior) | | | 92% (Superior) | | | 92% (Superior) | | | 92% (Superior) | | | 92% (Superior) | | | 92% (Superior) | | | 92% (Superior) | | | 92% (Superior) | | | | 92% (Superior) | | |
| **SAM Literacy Demand (Reading Grade Level)** | 85% (Superior) | | | 85% (Superior) | | | 85% (Superior) | | | 85% (Superior) | | | 85% (Superior) | | | 85% (Superior) | | | 85% (Superior) | | | 85% (Superior) | | | | 85% (Superior) | | |
| **SAM Literacy Demand (Writing Style)** | 40% (Adequate) | | | 40% (Adequate) | | | 40% (Adequate) | | | 40% (Adequate) | | | 40% (Adequate) | | | 40% (Adequate) | | | 40% (Adequate) | | | 40% (Adequate) | | | | 40% (Adequate) | | |
| **SAM Literacy Demand (Sentence Construction)** | 75% (Superior) | | | 75% (Superior) | | | 75% (Superior) | | | 75% (Superior) | | | 75% (Superior) | | | 75% (Superior) | | | 75% (Superior) | | | 75% (Superior) | | | | 75% (Superior) | | |
| **SAM Literacy Demand (Vocabulary)** | 75% (Superior) | | | 75% (Superior) | | | 75% (Superior) | | | 75% (Superior) | | | 75% (Superior) | | | 75% (Superior) | | | 75% (Superior) | | | 75% (Superior) | | | | 75% (Superior) | | |
| **SAM Literacy Demand (Road Signs)** | 80% (Superior) | | | 80% (Superior) | | | 80% (Superior) | | | 80% (Superior) | | | 80% (Superior) | | | 80% (Superior) | | | 80% (Superior) | | | 80% (Superior) | | | | 80% (Superior) | | |
| **Layout & Type** |  | | | | | | | | | | | | | | | | | | | | | | | | | | | |
| **SAM Graphic Illustrations, Lists, Tables, Charts (Front Page Graphics Show Purpose)** | 85% (Superior) | | | 80% (Superior) | | | 28% (Not Suitable) | | | 90% (Superior) | | | 70% (Superior) | | | 36% (Not Suitable) | | | 50% (Adequate) | | | 78% (Superior) | | | | 74% (Superior) | | |
| **SAM Graphic Illustrations, Lists, Tables, Charts (Type of Illustrations)** | 84% (Superior) | | | 83% (Superior) | | | 31% (Not Suitable) | | | 91% (Superior) | | | 69% (Superior) | | | 34% (Not Suitable) | | | 48% (Adequate) | | | 81% (Superior) | | | | 76% (Superior) | | |
| **SAM Graphic Illustrations, Lists, Tables, Charts (Relevance of Illustrations)** | 86% (Superior) | | | 82% (Superior) | | | 29% (Not Suitable) | | | 89% (Superior) | | | 71% (Superior) | | | 35% (Not Suitable) | | | 52% (Adequate) | | | 79% (Superior) | | | | 73% (Superior) | | |
| **SAM Graphic Illustrations, Lists, Tables, Charts (Graphics: Lists, Tables, Charts, Forms)** | 85% (Superior) | | | 81% (Superior) | | | 30% (Not Suitable) | | | 90% (Superior) | | | 68% (Superior) | | | 33% (Not Suitable) | | | 51% (Adequate) | | | 80% (Superior) | | | | 75% (Superior) | | |
| **SAM Graphic Illustrations, Lists, Tables, Charts (Captions to Explain Graphics)** | 84% (Superior) | | | 83% (Superior) | | | 29% (Not Suitable) | | | 92% (Superior) | | | 70% (Superior) | | | 36% (Not Suitable) | | | 49% (Adequate) | | | 79% (Superior) | | | | 74% (Superior) | | |
| **SAM Layout and Typography (Subheadings)** | 86% (Superior) | | | 82% (Superior) | | | 31% (Not Suitable) | | | 91% (Superior) | | | 72% (Superior) | | | 35% (Not Suitable) | | | 50% (Adequate) | | | 80% (Superior) | | | | 76% (Superior) | | |
| **SAM Layout and Typography (Typography)** | 85% (Superior) | | | 83% (Superior) | | | 28% (Not Suitable) | | | 89% (Superior) | | | 70% (Superior) | | | 34% (Not Suitable) | | | 48% (Adequate) | | | 81% (Superior) | | | | 75% (Superior) | | |
| **SAM Layout and Typography (Layout)** | 86% (Superior) | | | 80% (Superior) | | | 29% (Not Suitable) | | | 90% (Superior) | | | 71% (Superior) | | | 33% (Not Suitable) | | | 52% (Adequate) | | | 78% (Superior) | | | | 73% (Superior) | | |
| **Learning Stimulation & Motivation** |  | | | | | | | | | | | | | | | | | | | | | | | | | | | |
| **SAM Learning Stimulation & Motivation (Interaction Included in Text/Graphics)** | 56% (Adequate) | | | 51% (Adequate) | | | 31% (Not Suitable) | | | 90% (Superior) | | | 64% (Adequate) | | | 26% (Not Suitable) | | | 31% (Not Suitable) | | | 51% (Adequate) | | | | 54% (Adequate) | | |
| **SAM Learning Stimulation & Motivation (Desired Behavior Patterns)** | 54% (Adequate) | | | 49% (Adequate) | | | 29% (Not Suitable) | | | 89% (Superior) | | | 66% (Adequate) | | | 24% (Not Suitable) | | | 30% (Not Suitable) | | | 49% (Adequate) | | | | 55% (Adequate) | | |
| **SAM Learning Stimulation & Motivation (Motivation)** | 55% (Adequate) | | | 50% (Adequate) | | | 30% (Not Suitable) | | | 91% (Superior) | | | 65% (Adequate) | | | 25% (Not Suitable) | | | 29% (Not Suitable) | | | 50% (Adequate) | | | | 56% (Adequate) | | |
| **Cultural Appropriateness** |  | | | | | | | | | | | | | | | | | | | | | | | | | | | |
| **SAM Cultural Appropriateness (Logic, Language, Experience)** | 96% (Superior) | | | 81% (Superior) | | | 61% (Adequate) | | | 82% (Superior) | | | 61% (Adequate) | | | 36% (Not Suitable) | | | 41% (Adequate) | | | 66% (Adequate) | | | | 64% (Adequate) | | |
| **SAM Cultural Appropriateness (Cultural Image and Examples)** | 94% (Superior) | | | 79% (Superior) | | | 59% (Adequate) | | | 79% (Superior) | | | 59% (Adequate) | | | 34% (Not Suitable) | | | 39% (Adequate) | | | 64% (Adequate) | | | | 66% (Adequate) | | |
| **SAM Cultural Appropriateness (Suitable for Your Population)** | 95% (Superior) | | | 80% (Superior) | | | 60% (Adequate) | | | 79% (Superior) | | | 60% (Adequate) | | | 35% (Not Suitable) | | | 40% (Adequate) | | | 65% (Adequate) | | | | 65% (Adequate) | | |
| **TOTAL SAM Scores for Suitability of Information** | **81.25% (Superior)** | | | **75% (Superior)** | | | **42% (Adequate)** | | | **84.25% (Superior)** | | | **70% (Superior)** | | | **44 % (Adequate)** | | | **48.75 % (Adequate)** | | | **67.5 % (Adequate)** | | | | **68.75 % (Adequate)** | | |

- **Notes for Clarification**: The platform with the higher download count is marked with an asterisk (*) and was used for evaluation. Only one version of each app was included in the analysis, even if available across platforms, to avoid duplicate evaluation.
